# New perspectives on extracorporeal life support: expert teams and precise selection of candidates are transforming pediatric cancer and hematopoietic cell transplantation care

**DOI:** 10.3389/fonc.2025.1588403

**Published:** 2025-06-18

**Authors:** Caitlin Hurley, Matteo Di Nardo, Matthew Rees, Diego R. Hijano, Ahmed Said, Akshay Sharma, Lama Elbahlawan, Melissa R. Hines, Jennifer A. McArthur, Hitesh Sandhu, Saad Ghafoor

**Affiliations:** ^1^ Department of Pediatric Medicine, Division of Critical Care Medicine, St. Jude Children’s Research Hospital, Memphis, TN, United States; ^2^ Department of Bone Marrow Transplantation and Cellular Therapy, St. Jude Children’s Research Hospital, Memphis, TN, United States; ^3^ Pediatric Intensive Care Unit, Bambino Gesù Children’s Hospital, IRCCS, Rome, Italy; ^4^ Department of Oncology, St. Jude Children’s Research Hospital, Memphis, TN, United States; ^5^ Department of Infectious Diseases, St. Jude Children’s Research Hospital, Memphis, TN, United States; ^6^ Department of Pediatrics, University of Tennessee Health Science Center, Memphis, TN, United States; ^7^ Division of Pediatric Critical Care Medicine, St. Louis Children’s Hospital, Washington University School of Medicine, St. Louis, MO, United States; ^8^ Institute for Informatics, Washington University School of Medicine, St. Louis, MO, United States; ^9^ Department of Critical Care, Le Bonheur Children’s Hospital, Memphis, TN, United States; ^10^ Extracorporeal Therapies Service Line, Le Bonheur Children’s Hospital, Memphis, TN, United States

**Keywords:** extracorporeal life support (ECLS), extracorporeal membrane oxygenation (ECMO), pediatric oncology, pediatric hematopoietic cell transplant, pediatric critical care

## Abstract

Extra Corporeal Life Support (ECLS) for pediatric oncology and stem cell transplant patients over the past two decades has made progress. Substantial improvements in ECLS, Continuous Renal Replacement Therapy (CRRT), and mechanical ventilation techniques, along with enhanced anticoagulation management and infection control, have contributed to better patient outcomes. Additionally, advancements in HLA matching, donor selection, and the management of chemotherapy and transplant complications have further improved survival rates. The authors propose establishing an expert team and a standardized process to evaluate ECLS candidacy, addressing past controversies and optimizing outcomes for this vulnerable population. The criteria for candidacy have evolved significantly, necessitating expert evaluation.

## 1 Introduction

Survival of pediatric cancer has substantially improved over the last 2 decades, with a 5-year overall survival of ~ 80% largely due to enhanced diagnostics, novel treatment regimens, targeted therapies with reduced toxicities, and improved supportive care and management of treatment-related complications, including those that occur after hematopoietic cell transplantation (HCT) (1). Despite these advances, the survival of pediatric patients with cancer requiring critical care support remains lower than that of the general pediatric intensive care unit (PICU) patients (2). Nevertheless, recent advances in critical care management, such as early PICU admissions, careful use of noninvasive ventilation, lung-protective mechanical ventilation for intubated patients, and timely diagnostics for infections, have improved survival for PICU patients, including immunocompromised patients (2, 3). Additionally, advances in extracorporeal life support (ECLS) circuity and anticoagulation strategies in the general PICU population are improving ECLS outcomes and mitigating associated morbidity and mortality. Considering this current landscape in onco-critical care, we explored the evolving role of ECLS in caring for pediatric oncology and HCT patients, a population in which this intervention was once considered too high risk. Additionally, we share our current protocol for evaluating patient candidacy and facilitating referrals for ECLS.

## 2 Improving outcomes of children with cancer

Registry data from the American Cancer Society reveals that although the incidence of childhood cancer has increased, cancer-related deaths among children have decreased almost 70%: from 6.3 per 100,000 in 1970 to 1.9 per 100,000 in 2020 (4) A similar reduction occurred in adolescents (aged 13– <18 years), with cancer-related deaths declining 64% from 7.2 to 2.6 per 100,000 (4). Likely factors contributing to these improvements include large cooperative trials refining intensive multimodal therapies and advances in molecular profiling, which have enhanced prognostication and risk-adapted treatments (5). Additional scientific advances have facilitated the integration of targeted therapies, which have improved outcomes in cases where traditional approaches were ineffective (6–8). Furthermore, immunotherapy has become common for many high-risk or relapsed malignancies [e.g., dinutuximab for neuroblastoma and blinatumomab for B-cell acute lymphoblastic leukemia] and has greatly improved overall survival (6, 9–12). Despite these advances, treatment-related mortality remains a major cause of cancer mortality in children. More than 20% of childhood cancer deaths in high-income countries in the past 20 years have been attributed to treatment complications (13, 14). This finding underscores the ongoing need for better supportive care and cancer-directed treatments.

## 3 Improving outcomes of children after hematopoietic cell transplantation

The number of HCTs performed in the U.S. has nearly doubled over the past decade, reaching ~23,000 annually (15). Of those, ~1,300 are performed in children and young adults, with a modest annual increase (15, 16). Short- and long-term survival of children undergoing allogeneic HCT has improved concurrently: short-term (100 days post-HCT) survival rose from 86% (2000–2009) to 93% (2020–2019), while long-term (3-year) survival increased from 60% to 74%(15). These results are attributed to advances in managing disease relapse and acute and chronic graft-versus-host disease (GVHD); preventing infections; and better supportive care during the peri-transplant period. During the first 100 days post-HCT, organ failure, infection, and relapse are the leading causes of death in pediatric patients, especially in those who receive transplants from haploidentical or unrelated donors (15). Beyond that period, relapse remains the predominant cause of death.

Although acute organ failure caused by chemotherapy-related toxicity or genetic predisposition can be challenging, interventions can be tailored to improve outcomes. Mortality rates for pediatric HCT patients requiring critical care support have dramatically decreased from nearly 80% in the late 1990s and early 2000s, to 20% overall (and 50% for those requiring mechanical ventilation) since 2010 (16–22). These improvements likely result from early intensive care unit (ICU) referrals and enhanced multidisciplinary treatment strategies.

GVHD poses major risks for treatment-related morbidity and mortality after HCT. However, over the past decade, the frequency and severity of GVHD has decreased, and HCT outcomes have improved. Enhanced donor/recipient matching using high-resolution HLA (human leukocyte antigen) typing increased the use of reduced-intensity regimens, and T-cell–depleted grafts or post-HCT cyclophosphamide have all contributed to decreasing transplant-related mortality (23–25).

Several strategies have also been employed to reduce the risk of relapse after HCT. Using HLA-mismatched donors not only expands the donor pool but also enhances the graft-versus-leukemia effect. Strategies to achieve deeper remission before HCT, the application of cellular therapies, and optimizing conditioning regimens while minimizing toxicity have collectively reduced relapse rates (26–30). Although many of these strategies were initially developed for adult patients, similar trends are evident in pediatric populations (31–34).

## 4 Advances in managing challenges that affect outcomes of pediatric oncology and HCT patients

### 4.1 Infections

Patients undergoing HCT face a heightened risk of infection and associated morbidity (16). Infections and chemotherapy exposure can exacerbate organ dysfunction post-HCT, necessitating critical care support, mechanical ventilation, dialysis, and in rare cases ECLS (35). Prompt diagnosis and treatment of infections is crucial for ECLS success and overall outcomes (36). Traditional culture-based diagnosis of infection is often hindered by the patient’s prior antimicrobial exposure (37, 38). Furthermore, prophylactic antimicrobials can mitigate viral, bacterial, and fungal infections; however, their use also can lead to infections of multidrug-resistant organisms (39–42).

Recent advances in diagnostic tests and therapeutic interventions have enhanced our ability to detect and treat infections. For example, clinical molecular diagnostics can identify microbial pathogens, often surpassing conventional diagnostic methods (43–45). These tests expedite results, improve yield, and reduce hospitalization and antibiotic usage (46–49). Panels may include resistance genes, facilitating prompt targeted therapies (50). Such tests can be performed on various samples, including blood, respiratory tract specimens, stool, cerebrospinal fluid, and tissue (51). Metagenomics next-generation sequencing shows promise for advanced diagnosis, especially in complex cases [e.g., meningitis or encephalitis] (52).

Novel therapeutic agents, such as letermovir and maribavir for cytomegalovirus infections and novel beta-lactams combined with beta-lactamase inhibitors for multidrug-resistant bacterial infections, are increasingly utilized in cases where conventional treatments are limited by toxicity or resistance (53–55). Unfortunately, the current pipeline for clinical antibacterial drugs is limited in scope and innovation; thus, it inadequately addresses the growing threat of antibiotic-resistant bacteria (56–58). Nevertheless, advances continue to improve survival of these patients.

### 4.2 Acute kidney injury

Children with hematologic malignancies are susceptible to renal dysfunction, which is often described as acute kidney injury (AKI). Patients with tumor lysis syndrome, especially those with hyperphosphatemia, are at increased risk of AKI (59, 60). Severe tumor lysis syndrome is a relatively common indication necessitating ICU admission for frequent monitoring of renal function and metabolic status. In a cohort of 222 children with tumor lysis syndrome, 20 (9%) required CKRT, all of whom survived and regained renal function (60).

AKI is also prevalent in critically ill children after HCT; the incidence ranges from 21% to 84%, and severe AKI occurs in 12% (61). The etiology of AKI is often multifactorial, involving nephrotoxicity caused by medications (e.g., chemotherapy or antimicrobials), compromised renal perfusion due to capillary leak caused by hyperinflammatory states, sepsis, and transplant-related complications, such as sinusoidal obstruction syndrome and thrombotic microangiopathy (62).

Children with AKI have an increased risk of mortality. In a cohort of 484 post-HCT patients, AKI developed in 38%, and severe AKI developed in 42% (60). The relative risk of death is 4.6 times higher in patients with severe AKI, and ~30% of the severe AKI group required CKRT (61, 63).

Historical data indicate that survival of patients requiring CKRT is 26% to 33% (64). In a recent study of 68 pediatric HCT patients requiring CKRT, survival was 54% (65). In a cohort of children with severe sinusoidal obstruction syndrome post-HCT who underwent CKRT, 62% survived to ICU discharge (66). Enhanced survival is likely tied to better recognition of the adverse effects of fluid overload and the earlier initiation of CKRT when fluid overload exceeds 10%. Early detection and prevention of AKI progression are critical for improving outcomes. Tools such as electronic medical record alerts, renal injury risk–stratification systems (e.g., renal angina index), and biomarkers (e.g., cystatin C, kidney injury molecule-1, and neutrophil gelatinase-associated lipocalin) can aid in the early detection of AKI (62, 67).

### 4.3 Respiratory failure

Respiratory failure in the pediatric oncology and HCT population commonly results in PICU admission and contributes to morbidity and mortality (1, 22, 68, 69). Key treatment strategies for this complication include early recognition, lung-protective ventilation, and minimizing patient/ventilator asynchrony (70). Newer technologies, such as proportional assist ventilation (PAV), neurally adjusted ventilatory assist (NAVA), and airway pressure release ventilation (APRV), also improve invasive mechanical ventilation (71, 72). Informed decision-making based on a better understanding of physiological principles, underlying pathobiology, and treatment modalities has also improved treatment (73–76). Enhanced diagnostic capabilities, including Pediatric Early Warning Scores, and advanced tools, like computed tomography scans, bronchoalveolar lavage, and lung biopsies, have facilitated timely interventions, and comprehensive management strategies, such as the Pediatric Acute Lung Injury Consensus Conference version 2, have guided clinical practice (21, 77–88). Early ICU referrals enhanced multidisciplinary strategies, and more knowledge about the mechanisms underlying lung injury, including biotrauma and stress, have further informed clinical management (71, 72, 89–93).

Advanced monitoring, such as real-time measurements of volumetric CO_2_, dead space, and surrogates for work of breathing (e.g., esophageal manometry, airway occlusion pressure, and diaphragm thickness), have substantially enhanced our ability to optimize ventilatory support (94–99). Prone positioning has also emerged as a safe intervention in pediatrics and an essential intervention that improves oxygenation and overall outcome in adults (100–104). Ongoing multicenter trials, including the Protocol for the Prone and Oscillation Pediatric Clinical Trial [PROSpect; NCT: 03896763], are refining therapeutic strategies involving high-frequency oscillatory ventilation (HFOV) and prone positioning to further enhance patient care (105).

Ancillary therapies for both restrictive and obstructive respiratory failure in pediatric patients have been improved. A better understanding of fluid dynamics and clinical management strategies, including the use of diuretics and continuous kidney replacement therapy (CKRT), has been crucial in optimizing care (106–108). Additionally, therapeutic interventions, such as surfactant administration, inhaled nitric oxide, neuromuscular blockade, lung recruitment maneuvers, bronchodilators, immune modulation, corticosteroids, and ECLS, have expanded the therapeutic arsenal in pediatric patients with respiratory failure (88, 104, 109–115).

Looking ahead, the potential for personalized therapies is promising, with the integration of novel endothelial, genomic, and epigenetic biomarkers to assess the severity of respiratory failure (116–119). Research on molecular signaling pathways and fluid channel mechanisms may enhance our understanding of respiratory failure and identify new therapeutic targets (120–122). Additionally, mesenchymal cell–based therapies show promise for improving outcomes of pediatric respiratory failure (123). Leveraging these advances, the field is positioned to transform the landscape of pediatric respiratory failure, thereby paving the way for more precise, effective interventions to optimize care and outcomes of pediatric oncology and HCT patients.

## 5 Advances in ECLS for pediatric oncology and HCT patients

The improved approaches to treating the complications described above have rekindled interest in advanced life support, including ECLS, which was previously discouraged for pediatric oncology patients. Despite technological advances in ECLS pumps and circuits and improved anticoagulation strategies, evaluating ECLS candidacy in children with cancer remains complex (115, 124–126). This complexity arises from prognostic uncertainties related to the underlying malignancy, heightened risk of infection, and severe ECLS-related complications, such as bleeding and thrombosis, which are more severe in pediatric patients with cancer than in the general PICU population (127).

Current literature on ECLS candidacy among pediatric patients with cancer is limited and relies primarily on retrospective studies, case series, and anecdotal accounts (127). Many of those studies lack detailed data on the type of cancer, therapeutic exposures, and relevant variables crucial for clinical evaluations (128).

Over the past 2 decades, several pediatric oncology patients with cardiorespiratory failure that did not respond to comprehensive multimodal medical therapy have been supported by ECLS, yielding variable outcomes (129–135). A recent systematic review reported that the mortality rate of pediatric patients with cancer who underwent ECLS was comparable to that of the general PICU population who underwent ECLS: 60% *vs.* 55%, respectively, with inconsistent reporting of complications across studies (127). This elevated mortality can be attributed to the immunocompromised state, reduced organ reserve, and overall fragility of the patients with cancer. Additionally, studies have indicated a strong correlation among respiratory failure severity, mechanical ventilation duration, and mortality rates on ECLS (126, 136, 137).

Although bleeding complications are more prevalent in patients with cancer on ECLS, the incidence of nosocomial infections is similar to that in immunocompetent patients (138). Increased bleeding risk may arise from coagulopathy and thrombocytopenia associated with the underlying cancer and its treatment, compounded by systemic anticoagulation during ECLS (124, 133, 135). Alternatively, the prophylactic use of antimicrobials in immunocompromised patients with cancer may explain the comparable rates of nosocomial infections (138). Despite these findings, the lack of high-quality studies limits our ability to draw robust conclusions about the benefits of ECLS and its optimal timing in pediatric patients with cancer.

Patients undergoing HCT for malignant or nonmalignant disorders may also require critical care support. Over the last 2 decades, the probability of survival of HCT patients requiring PICU admission has improved to 48% to 75% (139, 140). This progress has renewed interest in using ECLS for HCT patients experiencing refractory cardiopulmonary failure (115).

Recent data indicate that survival of HCT patients supported by ECLS has gradually increased, though candidacy determination remains challenging (133–135, 141, 142). An analysis of the Extracorporeal Life Support Organization’s registry (1990–2020) revealed an increase in ECLS use among pediatric HCT patients and a substantial increase in survival to hospital discharge over the last decade (26% *vs.* 5%-10%) (142). Factors associated with higher mortality included the presence of malignancy, high peak pressures >30 during conventional mechanical ventilation before ECLS, and pulmonary or metabolic complications during ECLS (142). This increased survival is likely due to advances in HCT, critical care, and ECLS technology. For instance, adoptively transferring immune cells (e.g., virus-specific T cells) that target treatment-resistant viral infections during periods of immune reconstitution helps suppress life-threatening infections, and coating circuits and oxygenators with new anticoagulants (e.g., bivalirudin) helps mitigate bleeding risks (126, 143–145).

Recent guidelines indicate that ECLS may be considered for HCT patients with nonmalignant diseases or those with low risk of malignancy (re)occurrence at the time of ECLS evaluation (115). Additionally, the type of transplant (autologous *vs.* allogeneic) and the presence of active GVHD should be included in the ECLS candidacy evaluation (146–148).

During ECLS candidacy evaluations, clinicians must assess the reversibility of the critical illness, the extent of organ failure, transfusion dependence, bone marrow reserve, and the goals of care set by the patient and family (149–152). The importance of understanding improvements in cancer-directed and post-HCT care, as part of the ECLS candidacy decision and how it relates to the critical illness and the potential for recovery, is essential for ECLS success–the mortality risk due to ECLS-related complications increases by 1% to 3% per day of support (124, 153). In HCT patients, the number of organ failures and the need for invasive mechanical ventilation or renal replacement therapy are independent predictors of mortality that should be meticulously evaluated, as is the presence and response of GVHD to treatment (114, 149, 154).

### 5.1 Advances in managing anticoagulation during ECLS

Despite advances in ECLS technology since its inception in the 1970s, thrombotic and bleeding complications remain a substantial cause of morbidity and mortality (155). Managing anticoagulation in pediatric patients during ECLS poses a challenge–sufficient anticoagulation to prevent thrombosis must be balanced with coagulopathy to reduce bleeding. Historically, unfractionated heparin was the anticoagulant of choice (156). However, its reliance on antithrombin, the risk of heparin-induced thrombocytopenia, and unpredictable responses have spurred interest in using thrombin inhibitors as alternatives (157–160). Thrombin inhibitors show at least noninferior efficacy compared to unfractionated heparin without an increased risk of bleeding complications, and they offer simpler laboratory monitoring.

Additionally, literature on standardized blood product replacement thresholds that correlate with patient outcomes is lacking (161, 162). A recent systematic review highlighted these gaps, providing expert consensus recommendations, while identifying key areas for future research (163). The complexity of patient factors, underlying pathologies, and circuit variables in managing anticoagulation in pediatric patients during ECLS underscores the inadequacy of a one-size-fits-all approach. Biomedical informatics researchers are developing decision-support tools that incorporate multifaceted variables, thereby facilitating personalized, clinically applicable strategies to enhance patient outcomes.

### 5.2 Our process for assessing ECLS candidacy

As a quaternary pediatric hematology/oncology center lacking on-site ECLS capabilities, we developed a standardized protocol for early identification and transfer of high-risk patients. A collaborative intensivist team from St. Jude and Le Bonheur Children’s Hospital (LBCH) evaluates ECLS candidacy using defined clinical and oncologic parameters. Patients are stratified as “ECLS-unsafe” (those with irreversible organ injury or prolonged instability) or “ECLS watchers” (high-risk patients meeting specific respiratory/cardiac criteria) (**Table 1**). Through multidisciplinary review, the team considers three key factors: disease status and prognosis, treatment phase and future therapies, and comorbid conditions. Each case culminates in one of three determinations: immediate transfer, deferred transfer with monitoring, or non-candidacy (**Tables 2**–**4**).

**Table 1:** Triggers for determining “ECLS watcher” status.

Respiratory Triggers	Cardiac Triggers
Mechanical ventilation with FiO_2_ >60% for >6 hours OI >25 and/or OSI >18 (modified, severe PARDS definition) for >6 hours Acute escalation in respiratory support; initiation of iNO/HFOV DAH requiring inhaled TXA and/or rFVIIa Mediastinal mass with supplemental oxygen requirement Patient with status asthmaticus on mechanical ventilation	CPR >5 minutes within the last 24 hours (at the discretion of the ICU attending) Escalation to 2 or more inotropic/vasopressor agents, excluding milrinone and calcium, to maintain age-appropriate blood pressure Serum lactate >4 mmol/L or 2 sequential serum lactate levels >2.2 mmol/L (not due to leukemia or another cancer-producing lactate) Arrhythmia with hemodynamic instability within the last 24 hours Mediastinal mass compromising cardiac function 15%-20% decrease in EF from baseline or EF<40% with CRS

CPR, cardiopulmonary resuscitation; CRS, cytokine release syndrome; DAH, diffuse alveolar hemorrhage; EF, ejection fraction; FiO_2_, fraction of inspired oxygen; HFOV, high-frequency oscillatory ventilation; ICU, intensive care unit; iNO, inhaled nitric oxide; OI, oxygenation index; OSI, oxygenation saturation index; PARDS, pediatric acute respiratory distress syndrome; rFVIIa, recombinant activated factor VIIa; TXA, tranexamic acid

**Table 2:** ECLS candidacy considerations.

Complication	Threshold for ECLS consideration
Reversible respiratory failure	Oxygenation failure with rapidly worsening OI or an OI >25 for 24 hours. Ventilation failure defined as the inability to maintain pH >7.2 (respiratory acidosis), despite maximal CMV/HFOV support
Reversible hemodynamic instability	Inability to maintain age-appropriate blood pressure despite optimal fluid resuscitation Need for 2 or more high-dose inotropic/vasopressor agents (epinephrine or norepinephrine 0.1 μg/kg/min, dopamine 10 μg/kg/min, 10mg/kg/hr 10 mg and/or vasopressin 1 mU/kg/min) Rising serum lactate level or the presence of end organ dysfunction
Reversible heart failure	Reversible decreased LV/RV systolic function and/or presence of pulmonary hypertension or diastolic dysfunction St. Jude PICU or LBCH heart failure team will discuss patient with ECLS leadership prior to transfer VAD and/or HCT candidate
Reversible chemotherapy/treatment-related CRS	Course of action and potential for survival are ideally known Reversible chemotherapy-related cardiorespiratory failure
Newly diagnosed or recurrent mediastinal mass compressing the airway and/or major vessels	Ongoing treatment and therapeutic goals If possible, aim for nonemergent transfers and procedures for these patients.

CMV, cytomegalovirus; CRS, cytokine release syndrome; ECLS, extracorporeal life support; HCT, hematopoietic cell transplantation; HFOV, high-frequency oscillatory ventilation; LBCH, Le Bonheur Children’s Hospital; LV/RV, left ventricle/right ventricle ratio; OI, oxygenation index; PICU, pediatric intensive care unit; VAD, ventricular assist device.

**Table 3:** ECLS contraindications.

Absolute contraindications for ECLS in hematology-oncology patients	Relative contraindications for ECLS evaluated on a case-by-case basis
Presence of advanced directives–withdrawal of care/DNR Ongoing CPR or recent CPR with poor or absent neurological exam Lack of consent Bleeding despite maximal therapy Recent intracranial/intraventricular hemorrhage Moderate or severe static neurocognitive disease Presence of a genetic or metabolic syndrome with a poor prognosis	Primary St. Jude hematology-oncology team unable to round and care for the patient at LBCH Active viral/fungal infection despite adequate treatment Multiorgan failure Lack of expected recovery in cell counts or presence of pancytopenia/low white blood cell count HCT^1^ Prolonged mechanical ventilation >21 days Severe veno-occlusive disease Recent neurosurgical interventions, gastrointestinal interventions, or bleeds Uncontrolled diffuse alveolar hemorrhage High-risk malignancy

^1^See separate section for hematopoietic stem cell transplantation.

CPR, cardiopulmonary resuscitation; DNR, do not resuscitate; ECLS, extracorporeal life support; LBCH, Le Bonheur Children’s Hospital.

**Table 4:** ECLS considerations for HCT patients.

Patient comorbidities (organ reserve)
Primary disease indication for HCT: if oncologic, current disease status
HCT and graft considerations HCT-conditioning regimen Stem cell source HLA matching Autologous *vs* allogeneic transplantation Engraftment status (marrow function) Active infection(s) Refractory thrombocytopenia
HCT toxicities (TMA, GVHD, VOD, etc.)
Family and patients’ goals of care

GVHD, graft-*vs*-host disease; HCT, hematopoietic cell transplantation; HLA, human leukocyte antigen; TMA, thrombotic microangiopathy; VOD, veno-occlusive disease.

The transfer protocol emphasizes comprehensive preparation, beginning with early family counseling about ECLS indications. Clinical coordination includes direct physician-to-physician handoffs, complete medical record transmission with emphasis on medication reconciliation, and pre-transfer imaging review including vascular ultrasounds when indicated. Primary oncology teams maintain active involvement throughout the process. Absolute contraindications focus on irreversible organ damage, while relative contraindications incorporate disease-specific considerations for immunocompromised hosts.

Continuous process evaluation tracks multiple metrics: consultation frequency and outcomes, transfer timelines, ECLS utilization rates, and longitudinal patient outcomes. This systematic approach addresses the challenges of off-site ECLS availability while maintaining alignment with oncologic care priorities, demonstrating how specialized centers can develop effective partnerships to manage critical care needs for complex patient populations.

## 6 Conclusion

In recent years, advances in diagnostic and treatment capabilities have markedly improved outcomes for pediatric oncology and HCT patients. Enhanced infectious diseases management, anticancer treatments, HCT techniques, ventilation management, ECLS technology, precise anticoagulation, fluid management, and CKRT capabilities have all contributed to this progress. Improved understanding and clinical management of complications associated with chemotherapy and HCT have also played a crucial role. Consequently, outcomes for critically ill patients requiring ECLS have improved, making older data less relevant. However, determining ECLS candidacy for these patients remains a challenge.

We strongly advocate for the establishment of dedicated multidisciplinary teams comprising intensivists, ECLS directors, hematologist-oncologists, and infectious diseases specialists to make informed decisions about ECLS candidacy. These teams will benefit from cumulative experience, leading to continuous improvement in patient outcomes. St. Jude has witnessed substantial improvement in practice, understanding, and outcomes with the implementation of a formalized ECLS consultation team and process. Furthermore, collaboration between multidisciplinary teams from different institutions can facilitate the collection of granular prospective data, thereby advancing the standard of care for this vulnerable population and ensuring that the most critically ill pediatric patients receive the best-possible treatment. This collaborative approach is essential for advancing the field and improving the prognosis for pediatric oncology and HCT patients requiring ECLS. By identifying suitable candidates for ECLS, we can advance care and avoid futile measures.

## Data Availability

The original contributions presented in the study are included in the article/Supplementary Material. Further inquiries can be directed to the corresponding author.
