# Supplementary material for: New perspectives on extracorporeal life support: expert teams and precise selection of candidates are transforming pediatric cancer and hematopoietic cell transplantation care
Source: Front Oncol. 2025 Jun 18;15:1588403. doi: 10.3389/fonc.2025.1588403 (PMC12213495; doi:10.3389/fonc.2025.1588403)
Supplement: Supplementary file 1 [file DataSheet1.pdf]

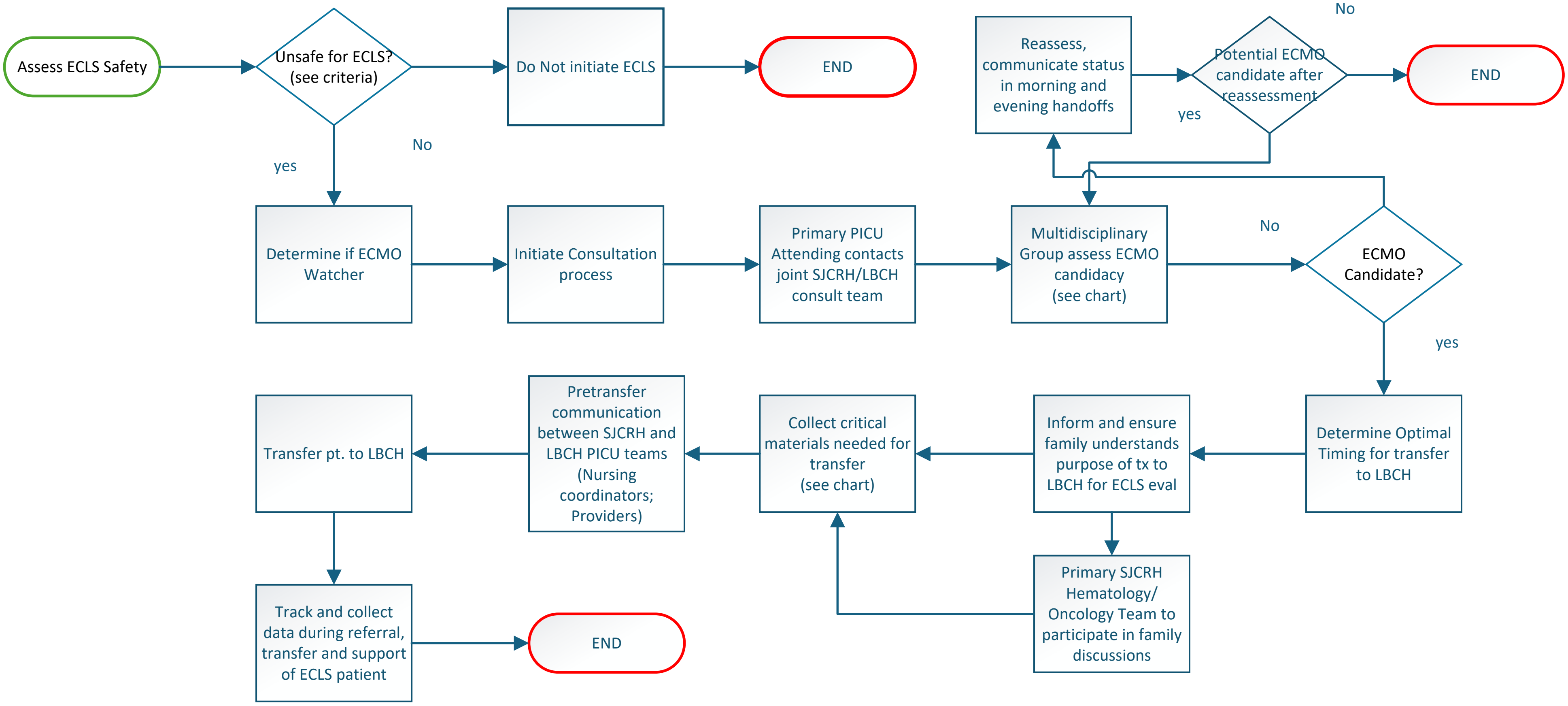

Criteria for Unsafe For ECLS:

- Actively undergoing cardiopulmonary resuscitation (requiring E-CPR)
- Significant hypoxic/ ischemic brain injury or renal dysfunction unrelated to need for ECMO
- Prolonged pre-ECMO hypoxemia (O2 saturations ,80% for >2 hours) or profound, sustained hypotension

Factors in Determining ECMO Candidacy:

- Oncologic factors
- Current disease status
- Expected 2-year oncologic survival
- Phase of current treatment
- Planned future therapies
- Additional comorbidities

Critical materials obtained and sent prior or at transfer:

- Relevant radiology images
- Discharge summary with completed med rec.
- Vascular ultrasounds of neck and lower extremities
- Additional assessments
